# Successful Continuation of Oral Intake in a Dysphagic and Tetraplegic Patient With Alternate Right and Left Complete Lateral Decubitus Positions in Rehabilitation

**DOI:** 10.7759/cureus.38667

**Published:** 2023-05-07

**Authors:** Yoshinori Maki, Mayumi Takagawa, Akio Goda, Junichi Katsura, Ken Yanagibashi

**Affiliations:** 1 Neurosurgery, Hikone Chuo Hospital, Hikone, JPN; 2 Rehabilitation, Hikari Hospital, Otsu, JPN; 3 Physical Therapy, Kyoto Tachibana University, Kyoto, JPN

**Keywords:** flexible endoscopic evaluation of swallowing, swallowing training, tetraplegia, aspiration pneumonia, lateral decubitus position, dysphagia

## Abstract

Cervical spinal cord injury can result in dysphagia and tetraplegia. Dysphagia therapy can be required to avoid aspiration pneumonia during oral intake for persons with cervical spinal cord injury. Complete lateral decubitus position may be a specific position for safe swallowing. However, the literature on dysphagia therapy in complete lateral decubitus position for persons with tetraplegia and dysphagia is limited. We present the case of a 76-year-old man with dysphagia and tetraplegia secondary to cervical cord injury. As the patient wished for oral intake, swallowing training in an elevated position of the head at 60° was already initiated. Two days after admission, aspiration pneumonia occurred. As the spasticity increased continuously, the patient could not comfortably undertake swallowing training in an elevated head position of 60°. The flexible endoscopic evaluation of swallowing (FEES) was performed for the patient. The patient did not swallow water or jelly safely in an elevated head position. However, the patient swallowed jelly safely in the right complete lateral decubitus position. Two months after the initiation of oral intake in the right complete lateral decubitus position, the second FEES revealed that the patient swallowed jelly and food in the form of paste safely in the left complete lateral decubitus position. To relieve the pain of the right shoulder induced by continuous right complete lateral decubitus position, the patient retained oral intake in the left or right complete lateral decubitus position alternately for six months without recurrent aspiration pneumonia. Right and left complete lateral decubitus positions when alternately performed in swallowing training can be useful and safe for a patient with dysphagia and tetraplegia secondary to cervical spinal cord injury.

## Introduction

Cervical spinal cord injury can result in tetraplegia and dysphagia [1,2]. The incidence of dysphagia secondary to cervical spinal cord injury can vary from 7% to 80% [1,3,4]. Dysphagia after cervical spinal cord injury can result in aspiration pneumonia, increasing morbidity and mortality [3,4]. To avoid unfavorable sequelae and to maintain nutrition in patients with tetraplegia and dysphagia related to cervical spinal cord injury, early detection and management of dysphagia seem essential [4]. Swallowing training for dysphagia secondary to cervical cord spinal injury seems not to be proposed as the gold standard, however, a tailor-made approach seems desirable to manage patients with tetraplegia dysphagia after cervical cord spinal injury [3].

Swallowing training is an established treatment in patients with dysphagia to improve oral intake of food and/or water and their quality of life [5]. Considering the risk for aspiration pneumonia in swallowing training, measures to prevent it are important. Swallowing training is usually performed while the patient is in a sitting position or with the head elevated. For patients with a high risk for aspiration pneumonia, a specific posture of the head or neck such as head rotation and chin-down position is combined with these positions to safely perform swallowing training [6-8]. However, swallowing training in a sitting or elevated position of the head or upper body can be challenging and even uncomfortable for patients who are unable to maintain these positions. Besides, when a patient is not able to rotate or move the neck due to hypertonia after cervical spinal cord injury, such a specific posture of the head or neck seems not suitable for swallowing training. Complete lateral decubitus position in swallowing training is an option for these patients; however, the literature on complete lateral decubitus position in swallowing training is limited [9,10]. As the complete lateral decubitus position still seems to be not standardized [9,10], this position introduced for dysphagia in persons with tetraplegia is not described to date. Herein, we report a case with dysphagia and tetraplegia after cervical cord injury in which the complete lateral decubitus position in swallowing training resulted in the successful introduction and continuation of oral intake. This study aims to propose a possibly safe position of continuous oral intake in a patient with dysphagia and tetraplegia.

## Case presentation

A 76-year-old man with dysphagia and tetraplegia was transferred to our department for rehabilitation therapy. Four months before, the patient slipped on the ground and hit his head and neck. Immediately after the injury, the patient sustained a cervical spinal cord injury due to a ground-level fall. Magnetic resonance images revealed cervical cord injury at the level of C5. Two weeks after the event, the patient had C5 complete tetraplegia. The patient also underwent tracheostomy due to residual right vocal cord paralysis and airway stenosis a month after the event. He was able to vocalize with a speech cannula. As he did not want tubal feeding or gastrostomy, oral intake in an elevated head position of 60° was already initiated at the previous institute for acute medical treatment. Oral intake of normal diet was continued in our hospital; however, aspiration pneumonia occurred two days after transfer to our hospital. Food residue was aspirated from the tracheostomy. Further, the patient mentioned discomfort due to the pain related to progressive hypertonia in the upper and lower extremities when he was taking food or water in an elevated head position of 60°. The position of the extremities gradually became adducted. Hypertonia was also observed in the neck. He also complained of pain in the shoulders, especially on the left side. Therefore, we planned the flexible endoscopic evaluation of swallowing (FEES). Based on the result of FESS, we modified the position during oral intake to reduce the risk of aspiration pneumonia.

Diagnosis and Rehabilitation Therapy

**Figure 1:**
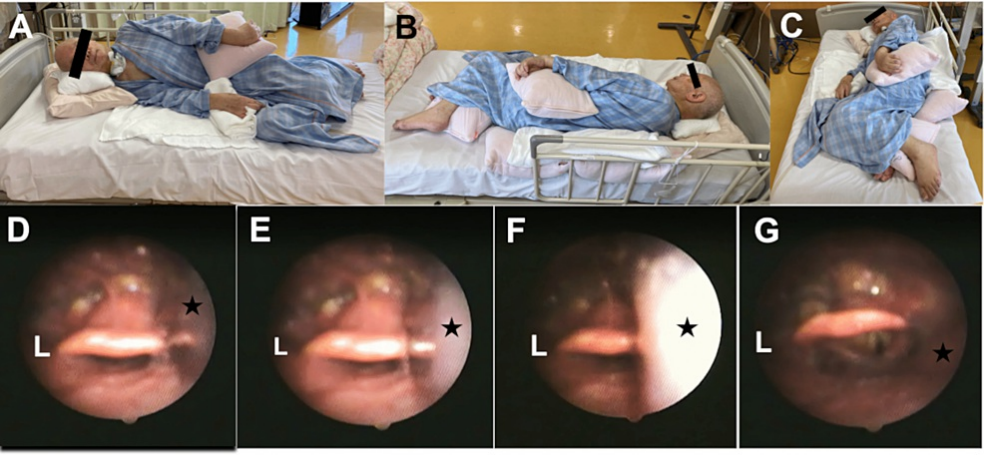
Right complete lateral decubitus position with the findings of swallowing videoendoscopy A to C: The images show the right complete lateral decubitus position. A cushion is placed under the patient’s head. The patient’s head is rotated slightly to the left side, and this position maximizes the space of the pharynx. Cushions and towels are placed to support the patient’s position and to relax the extremities of the patient. D to G: The images are of swallowing videoendoscopy. A jelly (black star) enters the right pharynx. The jelly bolus pools in the right pharynx, while the space of the right pharynx is maximized with the right complete lateral decubitus position. After the patient swallows the jelly, some of the boluses remain in the right pharynx, but the bolus does not enter the larynx L: Left

First, the patient was set in an elevated head position of 30° and was instructed to swallow blue-stained water and jelly, respectively, while undergoing the FEES procedure. However, both materials were not completely swallowed and residual materials in the vallecula of epiglottis were observed to enter through the pharynx and larynx to the trachea. Due to the hypertonia of the neck, the patient was not able to flex or rotate the neck. We considered that continuation of oral intake in an elevated head position of 30° increased the patient’s risk of aspiration. Subsequently, we attempted the complete lateral decubitus position for the patient. The patient was set in the right complete lateral decubitus position to not aggravate pain in the left shoulder. Cushions and towels were placed for the patient’s comfort and to rotate the head and neck slightly to the left (Figure 1 A-C). The patient was instructed to swallow jelly in this position. The jelly was observed to gradually pool in the right lateral wall of the pharynx, and the patient swallowed the bolus. Jelly residue was observed only slightly in the right lateral wall of the pharynx, but not in the larynx (Figure 1 D-G).

**Figure 2:**
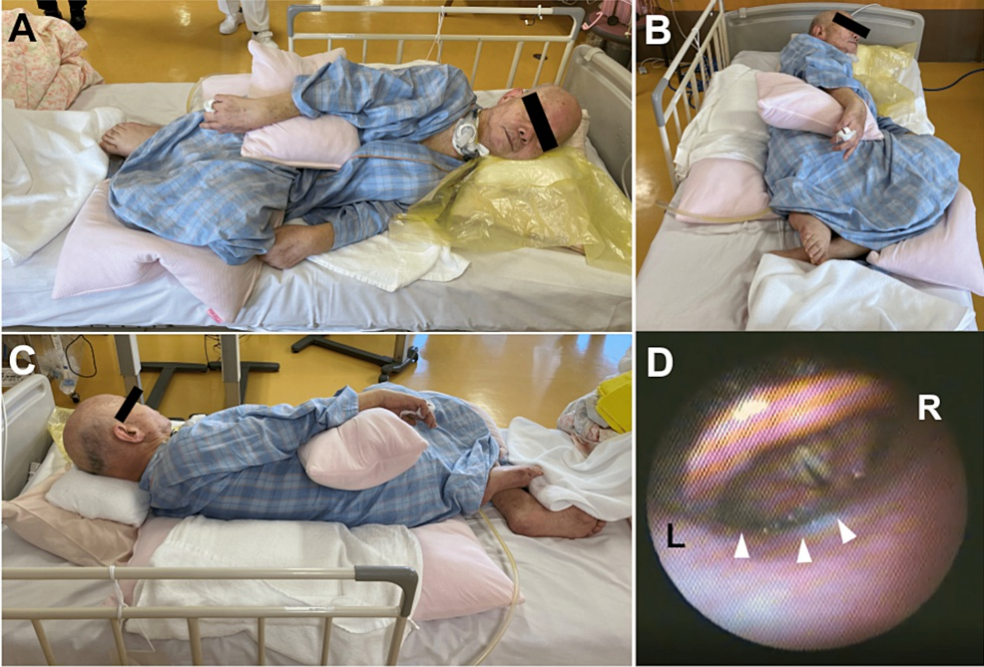
Left complete lateral decubitus position A to C: The patient is positioned in the left complete lateral decubitus position. Cushions and towels are also used in this position. D: Blue-stained water (white arrowheads) remains in the hypopharynx but does not enter the trachea L: Left, R: Right

The residue was removed with suction after the FEES. We determined that swallowing in the right lateral complete decubitus position could be safe for the patient. Oral intake in the right lateral decubitus position was continued for approximately two months without recurrent aspiration pneumonia. An oral medication was administered to relieve the pain in the left shoulder during this period, but the patient started to complain of pain in the right shoulder and upper extremity. We considered that pain in the right shoulder and upper extremity could have resulted from the continuous right complete lateral decubitus position. The FEES was used to re-assess the patient. The patient swallowed jelly in the right complete lateral decubitus position more smoothly in the second examination than in the first. Thereafter, the patient was set in the left complete lateral decubitus position (Figure 2 A-C). The patient was instructed to swallow blue-stained water. After swallowing, the blue-stained water remained in the piriform recess but did not enter the trachea (Figure 2 D).

Thus, the patient was instructed to swallow food in paste consistency after swallowing the blue-stained water. The food bolus did not enter the trachea either. Food residue was suctioned out appropriately. Though the patient was swallowing more smoothly in the right complete decubitus position than in the left complete decubitus position, we considered that swallowing in the left complete lateral decubitus position could also be safe for the patient. Following the second FEES, the patient continued oral intake approximately for 20 to 30 minutes a meal in the right or left complete decubitus position alternately. The patient was taking a total of 1217 kcal of jelly, rice gruel, liquidized food, and moisten minced food. After the patient started to orally take food and water in alternate complete lateral decubitus position, nutrition status ameliorated in four months as a blood examination revealed an increase in albumin and total protein from 2.9 to 3.0 g/dL, and 5.9 to 6.3 g/dL, respectively. At the end of every meal, the patient was instructed to take a spoon of thickened water to rinse away the food residuum [11]. The patient did not have recurrent aspiration pneumonia for six months. The shoulder pain was well controlled by taking two tablets of tramadol hydrochloride and acetaminophen daily. As the tracheostoma remained open, a speech cannula was used when the patient orally communicated. The patient was satisfied with the oral intake.

## Discussion

We described a case with tetraplegia and dysphagia following a cervical spinal cord injury. Aspiration pneumonia due to dysphagia occurred while our patient was orally taking food and water in an elevated head position of 60°. Therefore, oral intake in an elevated head position did not seem safe for our case. Alternate complete lateral decubitus position after the FEES successfully resulted in continuous oral intake in the patient. Though the following treatment is reported as rehabilitation management for patients with cervical spinal cord spinal injury such as swallow exercise, otolaryngologic surgery, and cough and respiratory muscle therapy [3], swallowing therapy under a specific posture like complete lateral decubitus position for patients with tetraplegia and dysphagia following cervical spinal cord injury seems not to be reported to date.

Complete lateral decubitus position in swallowing training was first proposed by Fukumura et al. in Japan [12]. They used this method in swallowing training for patients with dysphagia due to bulbar or pseudobulbar palsy secondary to cerebrovascular disease. With successful oral intake in the complete lateral decubitus position, Fukumura et al. showed an increase in the quality of life and activities of daily living of the patients [12]. Aspiration pneumonia can occur, prior to or following the swallowing reflex, when a food bolus falls through the pharynx and larynx to the trachea because of gravity [12]. In the complete lateral decubitus position, the patient lies on the lateral side of the trunk on the bed. This position facilitates the paste or water bolus to remain in the lateral wall of the oropharynx and hypopharynx due to gravity [11]. Fukumura et al. also reported using a skeleton model of the pharynx and larynx where approximately 14.2 mL of water pooled in the pharynx in the complete lateral position, whereas only 4.6 mL of water pooled in the sitting position. Thus, the food bolus can remain easily in the oropharynx and does not enter the larynx in the complete lateral decubitus position [12]. In addition, the direction of the food bolus to the larynx is perpendicular to gravity. As a result, swallowing in the complete lateral decubitus position can reduce the risk of aspiration pneumonia [12]. The residuum in the oropharynx can be rinsed away with 10 ml to 20 ml of thickened water [11], which was also effective in our case.

To date, the number of reports on complete lateral decubitus position for swallowing training seems to be limited in patients with dysphagia or healthy volunteers, especially in the English literature [9,10]. The technique of ensuring the complete lateral decubitus position is not sufficiently standardized and individualized for each case [13,14]. In our case, safe oral intake in the complete lateral decubitus position was successfully introduced for a patient with dysphagia and tetraplegia. Our case is the first report of right and left complete lateral decubitus positions alternately performed in swallowing training in this patient population. Due to increased hypertonia of the upper and lower extremities after cervical spinal cord injury [15], our patient complained of discomfort in the elevated position. In addition, our patient was not able to move the neck due to hypertonia either. Therefore, we thought that it was difficult to modify the position of the head and/or neck in the elevated position to establish safe oral intake for the patient.

Complete cervical spinal injury at C5 can reduce glottal adduction, vocal fold function, and cough reflex. These conditions can result in dysphagia and aspiration pneumonia in patients with cervical spinal injury [3,16,17]. Our patient had a high risk of aspiration pneumonia due to C5 complete cervical spinal injury and vocal cord paralysis. Aspiration pneumonia did occur while our patient was orally taking food and water in the elevated position. Therefore, we attempted oral intake in the complete lateral decubitus position after we performed FEES. Although the patient did not swallow jelly or water completely, the residue was located in the oropharynx or hypopharynx. The pharyngeal space was maximized with contralateral rotation of the head and neck to facilitate the residue pooling in the pharynx. The residue was appropriately rinsed away to prevent aspiration pneumonia. In addition to dysphagia, our patient had tetraplegia resulting in hypertonia of the upper and lower extremities. Hypertonia in our patient caused pain in the right shoulder while the patient was feeding orally in the right complete lateral decubitus position. To continue oral feeding comfortably, we had to relieve the pain of the patient, for which alternating laterality of the complete lateral decubitus position was effective. As the indication of swallowing training in the complete lateral decubitus position seems not to be established due to scant literature, the contraindication of this method seems not to be standardized either. However, we think this method may be contraindicated for patients with the following conditions: agitation due to dementia or cognitive impairment, pain induced by decubitus ulcer, and dysphagia due to esophageal or gastric problems.

As seen in our case, the complete lateral decubitus position in swallowing training can be additionally indicated with a combination of alternating laterality and postural modifications. A limitation of this study is that it is a single case report followed up over a short period. Therefore, further similar cases with long-term follow-up of outcomes are warranted. The tracheostoma in our patient remained open, which is considered a risk of dysphagia resulting in aspiration pneumonia in patients with cervical spinal cord injury [1]. It should be also addressed in further studies whether the complete lateral decubitus position in swallowing training can be effective to prevent aspiration pneumonia in patients with a residual tracheostoma.

## Conclusions

Although the complete lateral decubitus position in swallowing training seems not to be standardized as yet, this technique can be modified and adapted to each patient’s condition. In our case, right and left complete lateral decubitus positions performed alternately in swallowing training were useful for a patient with dysphagia and tetraplegia resulting from cervical spinal cord injury. Further studies are required to establish indications and contraindications of complete lateral decubitus positions in swallowing training.
